# Seeking Temporal Predictability in Speech: Comparing Statistical Approaches on 18 World Languages

**DOI:** 10.3389/fnhum.2016.00586

**Published:** 2016-12-02

**Authors:** Yannick Jadoul, Andrea Ravignani, Bill Thompson, Piera Filippi, Bart de Boer

**Affiliations:** Artificial Intelligence Lab, Vrije Universiteit BrusselBrussels, Belgium

**Keywords:** speech perception, temporal structure, rhythm, Bayesian, time series, autoregressive models, nPVI, timing

## Abstract

Temporal regularities in speech, such as interdependencies in the timing of speech events, are thought to scaffold early acquisition of the building blocks in speech. By providing on-line clues to the location and duration of upcoming syllables, temporal structure may aid segmentation and clustering of continuous speech into separable units. This hypothesis tacitly assumes that learners exploit *predictability* in the temporal structure of speech. Existing measures of speech timing tend to focus on first-order regularities among adjacent units, and are overly sensitive to idiosyncrasies in the data they describe. Here, we compare several statistical methods on a sample of 18 languages, testing whether syllable occurrence is predictable over time. Rather than looking for differences between languages, we aim to find across languages (using clearly defined acoustic, rather than orthographic, measures), temporal predictability in the speech signal which could be exploited by a language learner. First, we analyse distributional regularities using two novel techniques: a Bayesian ideal learner analysis, and a simple distributional measure. Second, we model *higher-order* temporal structure—regularities arising in an ordered *series* of syllable timings—testing the hypothesis that non-adjacent temporal structures may explain the gap between subjectively-perceived temporal regularities, and the absence of universally-accepted lower-order objective measures. Together, our analyses provide limited evidence for predictability at different time scales, though higher-order predictability is difficult to reliably infer. We conclude that temporal predictability in speech may well arise from a combination of individually weak perceptual cues at multiple structural levels, but is challenging to pinpoint.

## Introduction

To acquire a language, human infants must solve a range of intertwined inductive problems which, taken together, represent one of the most demanding computational challenges a child will ever face. One of the earliest and most basic of these component problems is to segment continuous speech into distinct units, such as words, syllables or phonemes. Segmentation problems recur at multiple levels of linguistic structure, and must be solved either before or in tandem with higher-level inferences or generalizations that are defined over these units (e.g., syntactic, morphosyntactic, and phonotactic rules). However, it is at present unclear—both theoretically and in terms of building speech technologies—which properties of speech allow this highly underconstrained inductive problem to be solved.

In this paper, we test whether this problem might be made more tractable by *predictability in the temporal structure* of speech. The key idea is that, if the timing of syllables follows any kind of pattern, this temporal pattern might be helpful for infants acquiring speech (Bialek et al., 2001; Nazzi and Ramus, 2003; Saffran et al., 2006) by providing infants with clues to predict where units begin and end (Trehub and Thorpe, 1989; Trainor and Adams, 2000). This hypothesis is corroborated by experimental evidence with adults: experiments in which simple artificial signals were taught to participants showed that when there was no temporal structure at all to the signals (i.e., signals just changed continuously over time), participants had a hard time learning to reproduce them (de Boer and Verhoef, 2012). This was true, even though the signals were based on vowels, and thus were recognizably speech-like. In an otherwise identical experiment, where signals did have clear temporal structure (i.e., there were regularly spaced building blocks separated by drops in volume), learning was much better even though the signals themselves were less speech-like (being produced with a slide whistle, Verhoef et al., 2014). Here we investigate the predictability of temporal structure of speech in a sample of 18 languages using three different statistical approaches. Specifically, we explore how well the occurrence of an upcoming syllable nucleus can be predicted on the basis of the times at which previous syllables occurred. In one of the three statistical models, we also test whether the previous syllable's intensity helps in predicting the time of occurrence of the next syllable.

We emphatically do not want to enter the debate about rhythmic classes of languages (stress-timed, syllable-timed, or mora-timed) and the ways to measure them. Much research has classified languages based on their temporal structure (Pike, 1945; Rubach and Booij, 1985; Port et al., 1987; Bertinetto, 1989; Fabb and Halle, 2012), reporting multiple acoustic correlates for language rhythmic class (Ramus et al., 1999; Patel and Daniele, 2003). Arvaniti (2012) has shown that many of the proposed measures are very sensitive to speaker, sentence type and elicitation method. In addition, she finds that groups of languages are classified differently by different measures, concluding that (p. 351) “any cross-linguistic differences captured by metrics are not robust […] making cross-linguistic comparisons and rhythmic classifications based on metrics unsafe at best.” Here, we investigate how durations and intensities of preceding syllables can help to predict the position and duration of a subsequent syllable, and whether more complex patterns than a simple fixed average duration play a role. Though to our knowledge there has been little investigation of higher-order timing structures in speech, it is clear that structure in higher-order timing patterns (e.g., at the sentence level) can influence processing of smaller units (e.g., syllables) in speech: for example, Reinisch et al. (2011) show that the timing of a preceding sentence can influence how people interpret stress in a subsequent word. Results like this suggest that complex timing patterns at multiple levels in speech are salient to listeners and influence processing, motivating our analysis of these patterns.

*Rhythm* in language is obviously more complex than just temporal predictability of syllables (e.g., involving the way stressed and unstressed syllables are grouped into feet, Goedemans and Van der Hulst, 2005). However, most of the existing notions of rhythm in speech depend on already having some knowledge of the sound system of the language. Our notion of predictability is therefore somewhat more basic than most notions of rhythm in the phonological literature. Going back to the origins of rhythm research in psychology (Bolton, 1894), we call *rhythmic* the temporal regularities in sound sequences and *rhythmical* those patterns of temporal intervals also containing variation in loudness. Bolton's very influential work (Bolton, 1894) has, on the one hand triggered much developmental work (e.g., Thorpe and Trehub, 1989; Trehub and Thorpe, 1989; Trainor and Adams, 2000), while on the other promoted empirical research on the relative importance of duration and intensity in segmenting general auditory input (Povel, 1984; Trainor and Adams, 2000; de la Mora et al., 2013; Toro and Nespor, 2015). Here, we put the emphasis on speech rhythmicity, rather than rhythmicality, hence testing the importance of durational information (rather than fine-grained spectral characteristics) in predicting future temporal regularities. In particular, we test whether the occurrence of syllable nuclei (characterized by peaks in intensity and maximum harmonics-to-noise ratio, i.e., voicedness) can be predicted from the (regularities in the) durations of the intervals between them. Therefore, we use only data about the syllable nuclei in our analysis.

In order to quantify the predictability of temporal structure in language, we investigated a small corpus of texts in 18 typologically and geographically diverse languages (listed in Table 1). We use a typologically and geographically diverse sample to exclude the possibility that temporal structure would somehow be an areal feature of Western European languages. As we are interested in the temporal structure of real speech, using word lists would not be useful, and therefore we use short stories. The example stories used in the illustrations of the IPA (International Phonetic Association, 1999) are ideal for this purpose. These are very short stories, either read from a text or spontaneously (but fluently) told. Although the stories are short this should not matter, because if rhythmic structure is to be of any use in acquisition, it should already be apparent from relatively short passages (Nazzi et al., 2000). Herein lies another difference with most existing literature on rhythmic measures: previous methods have been developed and used to quantify differences in rhythm between languages and hypothesized rhythmic classes (e.g., Arvaniti, 2012). Conversely, we are interested in the amount of temporal predictability that is present across languages, providing a set of clues to support the language learning process.

**Table 1:** **Information and numeric results for each language that was annotated and analyzed**.

**Language specs**				**Descriptive statistics**			**Distances and Distributions**		**Ideal Learner Predictions**			**ARMA Modeling**		
**Language**	**Language family**	**ISO**	**References**	**Number of nuclei**	**Number of rhythmic phrases**	**Median of distribution (ms)**	**Normality**	**nPVI**	**Estimated LR-INI mean**	**Estimated LR-INI variance**	**Differential entropy**	**(p,q) and differencing order of best ARMA**	**Size of akaike set**	**% Akaike weight taken up by *d* = 1**
Arabic	Afro-Asiatic	ara	Thelwall and Sa'Adeddin, 1990	211	15	173	0.07	38.6	0.02	0.25	0.72	(5,3),1	30	93.96%
Arrernte	Pama–Nyungan	aer	Breen and Dobson, 2005	220	18	177	0.074	35.4	−0.02	0.23	0.70	(1,1),1	21	98.89%
Cantonese	Sino-Tibetan	yue	Zee, 1991	123	20	144	0.076	26.8	0.06	0.11	0.35	(0,0),0	30	7.62%
Dutch	Indo-European	nld	Gussenhoven, 1992	159	18	148	0.078	45.9	0.04	0.33	0.87	(2,3),1	19	99.12%
Georgian	Kartvelian	kat	Shosted and Chikovani, 2006	173	18	164	0.113	47.8	0.01	0.41	0.98	(0,1),1	19	99.99%
Hindi	Indo-European	hin	Ohala, 1994	211	23	179	0.033	33.8	0.00	0.19	0.59	(0,1),1	34	91.14%
Hungarian	Uralic	hun	Szende, 1994	191	13	188	0.037	37.3	0.02	0.22	0.68	(0,1),1	50	65.05%
Igbo	Niger–Congo	ibo	Ikekeonwu, 1991	159	23	194	0.139	39	0.01	0.25	0.74	(0,1),1	16	100.00%
Italian	Indo-European	ita	Rogers and d'Arcangeli, 2004	185	20	185	0.056	41	0.04	0.27	0.76	(2,3),1	21	99.83%
Japanese	Japonic	jpn	Okada, 1991	187	24	131	0.163	49.2	0.10	0.35	0.90	(0,1),1	21	100.00%
Kunama	Nilo-Saharan	kun	Ashkaba and Hayward, 1999	185	41	196	0.078	41	0.09	0.28	0.80	(0,1),1	17	100.00%
Mapudungun	Araucanian	arn	Sadowsky et al., 2013	161	24	211	0.109	38.1	−0.01	0.25	0.73	(2,3),1	21	99.94%
Nuuchahnulth	Wakashan	nuk	Carlson et al., 2001	106	13	285	0.077	47.7	0.04	0.47	1.05	(0,1),1	21	99.99%
Spokane	Salishan	spo	Carlson and Esling, 2000	92	11	364	0.11	42.3	0.01	0.34	0.88	(1,1),1	20	100.00%
Tena Quichua	Quechuan	quw	O'Rourke and Swanson, 2013	238	36	249	0.112	42.3	−0.03	0.35	0.91	(0,3),1	22	98.43%
Thai	Tai–Kadai	tha	Tingsabadh and Abramson, 1993	181	33	251	0.064	41	0.02	0.33	0.87	(3,2),1	21	99.99%
Turkish	Turkic	tur	Zimmer and Orgun, 1992	169	14	159	0.055	32.9	0.00	0.17	0.54	(2,4),1	45	60.02%
Vietnamese	Austroasiatic	vie	Kirby, 2011	121	19	214	0.086	36.8	0.07	0.24	0.71	(0,1),1	19	98.19%

The left side of the table includes ethnographic information about the languages and descriptive statistics of our sample in terms of syllable and phrase structure. The right side of the table provides results for each language. For the first analysis, the correlation between the Kolmogorov-Smirnov D and nPVI measures can be noticed. Next, we present measures about an ideal learner's inference of the LR-INI (the logarithm of the relative INI lengths; see section Analysis and Results: Distributional Statistics of Temporal Structure (Order 1)), and the last three columns present the raw results of the ARMA analyses, including both the single best-fitting model as well as the results of calculating the Akaike weights and sets. Results from higher-order models should be interpreted keeping in mind the low predictive power of ARMA models for small sample sizes.

Story reading generally has a speaking style of its own. Analyses of Dutch (Theune et al., 2006), French (Doukhan et al., 2011), and Spanish (Montaño et al., 2013) show that compared to every-day speech, narrative speech tends to: (i) have more exaggerated pitch and intensity contours, (ii) be slower, (iii) have more pauses, (iv) include words with exaggerated pitch and intensity. Confusingly, in the literature this is often referred to as storytelling, but in fact most research is about stories that are read aloud from a prepared text. Spontaneously told stories have similar features, but more pauses and hesitations, and tend to have slower speaking rate (Levin et al., 1982). The features of story reading and storytelling are comparable to those of infant-directed speech (Fernald and Kuhl, 1987; Fernald et al., 1989), which facilitates word learning (Fernald and Mazzie, 1991; Filippi et al., 2014). Although story reading/telling style is therefore different from adult-adult dialog style, it may be more representative of the language intake (i.e., that part of the input that infants actually use in acquiring speech; Corder, 1967).

We use three increasingly sophisticated statistical techniques to quantify the predictability of syllable durations in our speech samples. The techniques make predictions based on increasingly long sequences, namely:

- Length 0: global distributional properties of the language determine when the next syllable will occur,
- Length 1: the time of occurrence of the next syllable is based on the previous syllable, i.e., there is a non-random distribution of relative duration of adjacent elements, or
- Length >1: when the next syllable will occur is based on the duration of multiple previous elements.

If temporal structure of speech is indeed predictable, this should be reflected in the outcome of our analyses. Our story-reading dataset might not be fully representative of ordinary adult-directed speech. However, the dataset is appropriate to look for temporal predictability, given the net-content of exaggerated features and comparability with infant-directed speech. If there is no structure at all to be found in this kind of speech, then there would be no reason to expect it in normal, less-controlled setting.

## Materials and methods

### Materials: corpus

The audio files were recordings of the narrative texts used in various publications of the international phonetic association, used as illustrations of the sound systems of different languages. Most often Aesop's fable “The North Wind and the Sun” is used for this purpose, but sometimes other (native) stories are used. Crucially, all of these transcriptions and recordings have been published in the Journal of the International Phonetic Association as part of the series of “Illustrations of the IPA” and a number are also available in the IPA handbook (International Phonetic Association, 1999). Sources per language are indicated in Table 1. The story consists of $177_{159}^{190}$ (median, first and third quartile) syllables divided over 5–13 sentences.

### Methods: annotations

**Figure 1:**
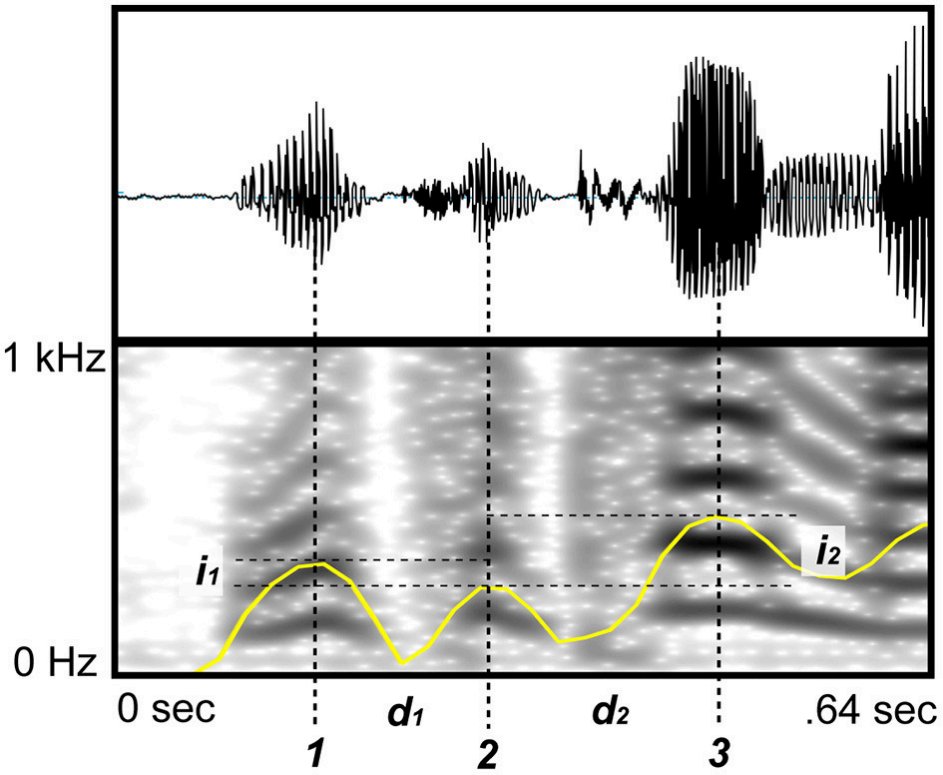
**Nuclei annotation methods**. The nuclei of each syllable (denoted by the corresponding syllable number 1,2,3,…) were annotated using acoustic information and visual information from the sound wave (top), the spectrogram (bottom, Fourier's window length equals 0.05 s), and the signal intensity (yellow curve). Distances between adjacent nuclei are denoted by *d*_*s*_ and *i*_*s*_ are the corresponding differences between intensities of adjacent nuclei.

The automatic methods for finding syllable centers we had available (Mermelstein, 1975; de Jong and Wempe, 2009) did not yield satisfactory results for the range of languages, speakers, speaking rates and speaking volumes that were in our sample, Hence we proceeded to annotate the sample manually. This has the advantage that our annotations represent the human-perceived syllable centers instead of computer-extracted data based on a predetermined set of features. Moreover, fine-tuning the parameters of automatic methods (Mermelstein, 1975; de Jong and Wempe, 2009) for each passage would introduce at least as much variability and subjectiveness as annotating the syllable centers manually. The centers of syllables were identified by ear, and their precise location was identified as the position where amplitude was highest and the harmonic structure was clearest (Figure 1). The transcriptions in the IPA articles were used to indicate phrase and sentence breaks, so that we could identify chunks of speech with uninterrupted rhythm. In addition, we indicated other points where the speaker paused and interrupted the rhythm. YJ re-checked all the cases where a break might have been forgotten, in order to have a more consistent dataset. Annotations were made in PRAAT versions 5.3.49–6.0.11 (Boersma and Weenink, 2013). Consistency between raters was ensured by having four of the considered languages annotated by two raters. The pairwise distance between all annotations was computed using Dynamic Time Warping (Sakoe and Chiba, 1978), a widely-used algorithm for aligning temporal sequences, where we use absolute time difference between two annotated nuclei as the distance metric. The sum of squared errors (i.e., sum of squared differences of matched annotated nuclei timings) between annotators for the same language was at least 10 times lower than the sum of squared errors between different languages or between real and randomly-generated annotations.

### Methods: mapping languages to durations

Having this set of annotated points in time for all languages, we then calculated the time distance between nuclei of adjacent syllables, i.e., the inter-nucleus-interval durations (INI), and the difference in intensity between those nuclei. Hence each language corresponds to two vectors *D = (d*_1_,*d*_2_,…,*d*_*n*_*)* and *I = (i*_1_,*i*_2_,…,*i*_*n*_*)* where *d*_*s*_ is the INI and *i*_*s*_ is the difference in intensity between syllables *s* + 1 and *s*, for *s* < *n* (Figure 1). Moreover, the indicated phrase breaks and pauses are used to discard the associated INIs. Note that these intervals are not removed from the time series, but replaced by a missing value (*NA*) that can be handled properly by each analysis.

It is mathematically convenient and cognitively plausible (Grondin, 2010; McAuley, 2010) to work with the logarithm of duration. This is cognitively plausible given Weber's (1834) law that the perceived differences between signals depend on the magnitude of the signals. It is mathematically convenient, because the *difference* between the logarithms is proportional to the *ratio* of the original numbers. The logarithm of the INIs therefore abstracts over absolute duration, accounting for variability in speed between speakers and over time: for example, for both fast and slow speakers, adjacent syllables with equivalent durations would lead to a difference of zero for the logarithms.

## Analysis and results: simple distributional measures (order 0)

We started by investigating distributional predictability in languages, namely whether information on presence and frequency of INIs provides information on the temporal organization of that language. We calculated the Kolmogorov-Smirnov D (Kolmogorov, 1933; Smirnov, 1948) statistic to quantify normality for each language. The D for each language is calculated as the difference between the empirical INI distribution for that language and a theoretical normal distribution with the same mean and standard deviation. We then tested how this measure relates to temporal variability by comparing it with a common measure of speech rhythm, the normalized pairwise variability index (nPVI, Grabe and Low, 2002). The nPVI is a measure of variability between adjacent durations, calculated as

$$\begin{array}{l} nPVI=\frac{100}{n-1}\overset{n-1}{\underset{t=1}{\sum }}|\left(d_{t}-d_{t+1}\right)/0.5\left(d_{t}+d_{t+1}\right)|, \end{array}$$

where *n* is the number of syllables, and the factor 100 normalizes the number to be between 0 and 100 (Patel and Daniele, 2003). A “metronomic language” composed of a series of similar INI will have a low nPVI (tending to zero as the INIs become identical). A language with strong temporal variability in INI, composed for instance of alternating short-long INI, will have high nPVI. Note that the nPVI measure here is calculated in a slightly different way than usually, based on INI lengths instead of the lengths of syllables.

**Figure 2:**
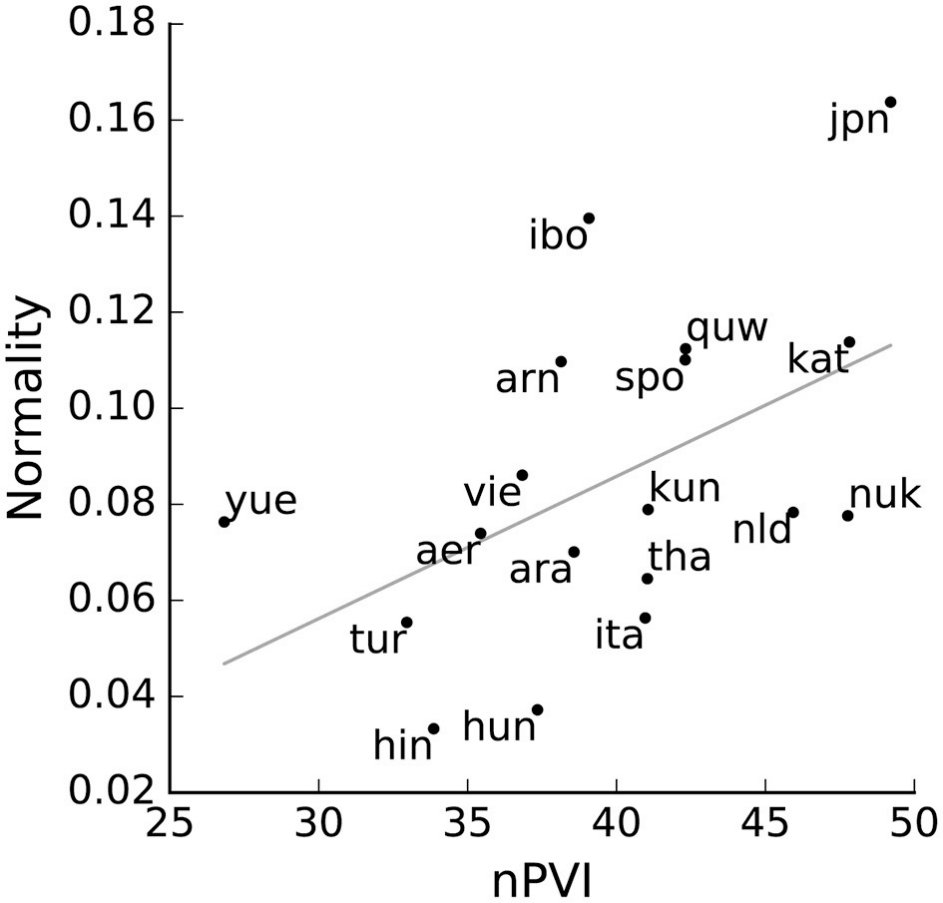
**Two ways of measuring rhythm variability**. For each language, the nPVI is plotted against our non-uniformity measure, showing a good correlation between the two (linear fit in gray).

We found a significant correlation between Kolmogorov-Smirnov D and nPVI (Spearman rank correlation = 0.60, *p* < 0.01, Figure 2). This high and positive correlation between our simple measure of normality (of order 0) and the more complex nPVI (which takes into account order 1 difference between syllables) shows that they both capture some common aspects of temporal structure of the signal. Our measure is possibly the simplest metric for temporal structure, suggesting that the complexity of nPVI adds little explanatory power to straightforward distributional measures. This analysis implies that most of temporal structure in a language as captured by a common measure of rhythmicity can be equally well judged by assessing whether syllable nuclei occur at normally distributed durations. Far from proposing one additional metric to quantify structural regularities in speech, we instead suggest that many existing metrics should be used carefully and critically, as they may embody very superficial features of speech (Loukina et al., 2011; Arvaniti, 2012).

For some languages, such as Thai, metrics are very different from those published in previous reports: the nPVI ranges between 55 and 60 in Romano et al. (2011) vs. our 41. In other languages, predictions are close: in Arrente one can compare our 35.4 with the range 39.6–51.2 found in Rickard (2006). Finally, for some languages we get almost identical numbers as in previous studies: for Italian, both our data and Romano et al.'s (2011) show nPVIs at 40 ± 1. Some issues about nPVI comparisons should be kept in mind. First, we purposely focussed on less-studied languages, and only some languages considered here had been analyzed at the level of rhythm and nPVI elsewhere. Moreover, for some languages several discordant measures of nPVI are available from different studies, making the selection of one previously-published nPVI per language quite arbitrary. In general, we do not find a strong association with previous studies probably because, as previously remarked (Arvaniti, 2012), values for the same rhythm metric applied to different corpora of the same language can vary a lot by study.

## Analysis and results: distributional statistics of temporal structure (order 1)

### Why use distributional methods?

We can make baseline inferences about temporal structure by quantifying the *distribution* of the logarithms of the ratio of adjacent INIs (i.e., the difference of the logarithm of adjacent INIs) observable among the languages in our sample. In the most temporally regular language, all adjacent syllables would have equal durations (i.e., equal INIs), and this distribution would be a point mass on 0 (the ratio between equal-length INIs is 1, whose logarithm is 0). In a language that has completely unpredictable temporal structure at this level, the duration of the preceding syllable provides no information about the duration of the following syllable, so this distribution would be uniform over a sensible range.

Standard tests for normality (D'Agostino and Pearson, 1973) suggest that we cannot reject the hypothesis that the data are drawn from an underlying Normal distribution for all 18 languages (at *a* = 0.05). As such it is reasonable to proceed under the assumption that the differences of the logarithm of the INIs are normally distributed. This assumption allows us to compute measures of predictability associated with normally distributed data. Many standard tools exist to estimate the shape of this distribution from a noisy sample, which is what our annotations represent. We calculated estimates for the mean μ and variance σ^2^ of this distribution for each language. Maximum a-posteriori (MAP) point-estimates (under an uninformative prior—see below) for all languages are shown in Table 1. The mean always centers around 0, and the average variance is around ¼ (0.28), suggesting a moderate level of predictability across languages.

### Bayesian inference for distributions of speech timing events: a primer

A more satisfying approach—utilizing all the information in the distribution—is to compute the full posterior distribution *P*(μ, σ|*R*) via Bayesian inference. This approach is useful in three respects. First, it provides a more complete picture of the structure in our data at this level. Second, experiments of perception and estimation of time intervals suggest humans process temporal regularities in a Bayesian fashion, where expectations correspond to a-priori probability distributions affecting top-down perception of incoming stimuli (Rhodes and Di Luca, 2016). Third, it provides a way to model the judgements of an ideal learner who observes these data: *what generalizations could an ideal learner infer from this evidence base?* In intuitive terms, the posterior distribution represents an ideal observer's updated beliefs after observing evidence and combining this information with the beliefs it entertained before observing the evidence (*prior* beliefs). The updated posterior beliefs are said to be *rational* or *ideal* if the particular way in which the learner combines prior beliefs and observed evidence follows the principles of conditional probability captured in Bayes' theorem. This way of modeling inference aligns with human learning in many domains (Griffiths et al., 2010), and provides a normative standard that quantifies how an evidence-base could be exploited by an ideal observer—which is exactly what we wish to achieve here. Standard techniques from Bayesian statistics (e.g., Gelman et al., 2004, p. 78) allow us to formulate an unbiased prior *P*(μ, σ) for the inductive problem at hand. Specifically, the Normal-Inverse-Chi-Square conjugate model (e.g., Gelman et al., 2004), with *k*_0_ = 0, *a*_0_ = 0, *v*_0_ = −1, for arbitrary μ_0_, ensures the prior is *uninformative*: in other words, the prior expresses uniform expectations about μ and σ^2^, so MAP estimates correspond to maximum likelihood estimates, and ideal learner predictions are unbiased.

**Figure 3:**
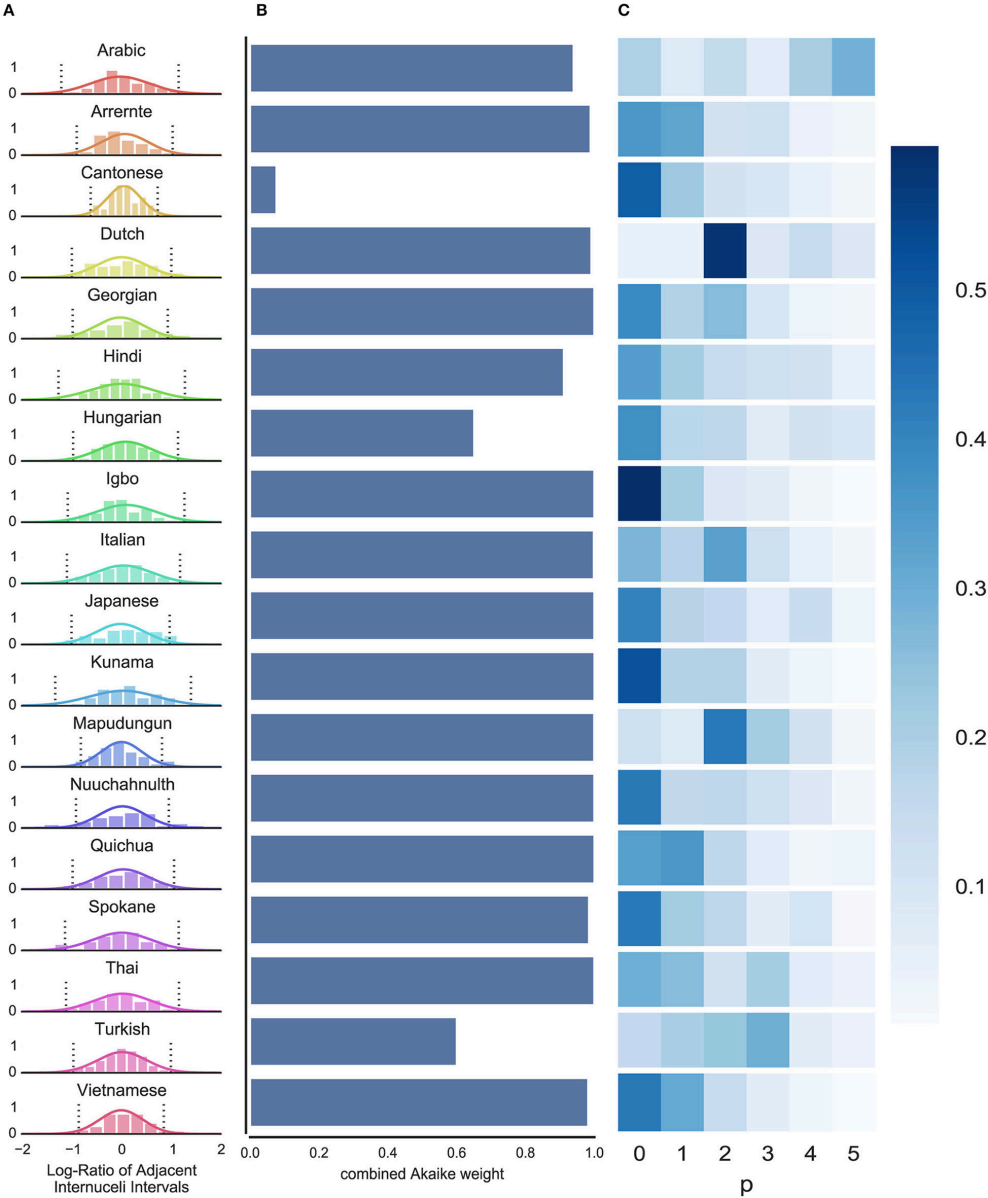
**Results of Bayesian and time series analyses**. **(A)** Distributions of the log-ratio of adjacent INIs for all languages: most languages have a wider spread, indicating less predictability; a few languages show a narrower distribution (e.g., Cantonese), indicating higher predictability at this level. Normalized histograms show the raw empirical data; Solid lines show the ideal learner predictions; Dashed lines show 95% confidence intervals for the ideal learner predictions. **(B)** The proportion of Akaike weights taken up by models that use the ratio between subsequent INI lengths (differencing order *d* = 1; as opposed to the absolute lengths, *d* = 0) shows that, in the vast majority of language samples, the relative length data provide a better ARMA fit (cfr. last column in Table 1, % Akaike weight taken up by *d* = 1). **(C)** The accumulated Akaike weights of all fitted ARMA models for each AR-order p do not show a clear picture of a predominant order of the ARMA model providing the best fit.

The posterior *P*(μ, σ|*R*) can be derived analytically under this model. We interrogate this posterior for targeted measures of predictability. For example, we can quantify the *degree of predictability* available to an ideal learner who is exposed to a temporal sequence of syllables: we model a learner who encounters these data, induces estimates of μ and σ^2^ via Bayesian inference, and goes on to use those estimates to make predictions about the time of occurrence of future syllables. In Bayesian statistics, the distribution describing these predictions is known as the *posterior predictive* distribution, and can be calculated exactly in this model. Our analysis pipeline assumes the learner induces estimates for μ and σ^2^ by drawing a random sample from their posterior, and makes predictions by drawing random samples from the Normal distributions defined by those estimates. To account for the randomness which underpins the learner's sampled estimates of μ and σ^2^, the model integrates over the posterior for these parameters, computing predictions under each parameter setting, and weighting those predictions by the posterior probability of those parameters given the data (and the prior). Even under an unbiased prior, this is a meaningful operation since it takes into account inferential uncertainty about μ and σ^2^, and propagates that uncertainty through to the model's predictions. In this respect, the model's predictions are conservative by admitting variance in predictions (compared to, for example, predictions computed under maximum likelihood estimates of μ and σ^2^). The specific form of the posterior predictive distribution in this model is Student's t. More formally, it can be shown that:

$$\begin{array}{lll} p\left(r^{new}|R,F\right) & = & \int \int p\left(r^{new}|\mu ,\sigma^{2}\right)p\left(\mu ,\sigma^{2}|R,\Phi \right)\;d\mu \;d\sigma \\ = & t_{n-1}\left(\bar{r},s\right), \end{array}$$

where *r*^*new*^ is the new interval to be estimated, *n* is the number of data points observed, Φ are the parameters of the prior specified above, $\bar{r}$ is the mean of the observed data *R*, and $s=\left(1+n\right)\sum {\left(r_{i}-\;\bar{r}\right)}^{2}\;/n(n-1)$. The second line of this equation reflects a standard result in Bayesian statistics (see Gelman et al., 2004). We computed these distributions for each language: Figure 3A shows these predictions, superimposed on (normalized) histograms of the raw data *R*.

### Bayesian inference in our dataset: results and discussion

The similarities between these distributions across languages are intuitively clear from Figure 3A. We provide a quantitative measure of structure. Though various appropriate measures are available, we report the information-theoretic *differential entropy* of these predictive distributions, which is a logarithmic function of the variance. Differential entropy directly quantifies the information content of an unbiased, ideal learner's predictions in response to distributional information on first-order temporal regularity. Formally, differential entropy is defined for this problem as follows:

$$\begin{array}{l} h\left(r^{new}\right)=\int p\left(r^{new}|R,\Phi \right)\log p\left(r^{new}|R,\Phi \right)dr^{new} \end{array}$$

Table 1 presents the differential entropy of the posterior predictive distribution, for each language. Lower values represent higher predictability: an ideal learner could make reliable predictions about the time of occurrence of an upcoming syllable in Cantonese, for example (entropy = 0.35), but would make less reliable predictions about Georgian (entropy = 0.98). In other words, in Cantonese more than Georgian, a few relative syllable durations provide information about the temporal structure of rest of the language.

We are hesitant to draw strong generalizations about predictability cross-linguistically from this small dataset. However, the distribution of predictability across the languages we have analyzed provides a window onto the variation in predictability we might expect. For example, the mean entropy across languages is 0.77; the lowest entropy is 0.3; and the highest entropy is 1.05. Of the 18 languages, 10 have entropy lower than this mean, and 14 have entropy lower than 0.9. Intuitively, this suggests most languages cluster around a moderate level of predictability at this level of analysis. Few languages are highly predictable (entropy → 0) or effectively unpredictable (entropy → ∞). An ideal learner who pays attention to these temporal regularities in speech will be better at predicting the location of the nuclei of an upcoming syllable than a learner who does not. Obviously, both hypothetical learners will still face uncertainty.

The ideal-learner analysis provides a range of tools for exploring learnability and predictability that could be generalized to more complex notions of temporal structure. The approach also offers potentially useful connections to language acquisition and inductive inference more generally. For example, in ideal-learner models of language acquisition, the prior distribution is often understood to represent inductive biases. These inductive biases, either learned or inherent to cognition, are imposed by the learner on the inferential problem. This perspective provides a framework to ask and answer questions about perceptual biases for temporal regularity. For instance, how strong the prior bias of a learner must be for her to reliably perceive high temporal regularity – over and above what is actually present in the data (Thompson et al., 2016). We leave these extensions to future work, and turn instead to higher-order sequential dependencies.

## Analysis and results: time series analysis for (higher order) sequential structural dependence

### Structure beyond metrics and distributions

Our previous analyses, in line with existing research, quantified rhythmic structure using minimal temporal information: first-order pairwise temporal regularities between adjacent syllables. Given the existing metrics and results for structure at this level (Arvaniti, 2012), and the inconsistency among associated findings, a natural alternative approach is to search for *higher-order* temporal structure, utilizing more features and a more complex statistical representation of the data. We address the question: does the preceding *sequence of N syllables* provide information about the timing of the upcoming syllable?

Structure at this level cannot be captured by typical first-order measures (e.g., Chomsky, 1956) employed in the literature (Arvaniti, 2012). In light of the disparity between intuitive impressions of rhythm in speech and empirical studies that fail to recover these intuitions (e.g., Dauer, 1983), perhaps this gap is made up in part by higher-order structural regularity, not visible to first-order methods. Specifically, we test whether sequential information about duration and intensity affects the predictability of future durational information. In other words: can we predict when the next syllable nucleus will occur, knowing the intensity and time of occurrence of the previous nuclei?

### ARMA: timing of occurrence of future nuclei as linear combination of past nuclei timing

Though there are many ways to model higher-order dependencies in sequences, a natural starting point is to approach the question using standard statistical tools from *time series analysis*. We model our data using a commonly used autoregressive moving-average (ARMA) process (Jones, 1980; Hamilton, 1994). In brief, an ARMA model tries to predict the next value in a time series from a linear combination of the previous values (see below for details). As explained in our introduction, the predictability of these timings may be beneficial during language acquisition: if the ARMA model is able to discover predictive regularities at this level, then in theory so could a language learner. In addition to the preceding INI lengths, we allow for an extra value (the difference in intensity of the previous syllable nucleus) to be taken into account in the prediction. Taking intensity into account in an ARMA model allows us to include a basic form of stress (in which intensity plays some role) in the predictions, which may be useful in languages where stressed and unstressed syllables alternate. Using this approach, we ask two questions: (i) is temporal predictability better captured by a linear relation between INIs or the same relationship between their *ratios*?; and (ii) is temporal predictability improved (with respect to zero- and first-order predictions) by basing predictions on more than just the single previous INI?

### ARMA for speech timing: a short introduction

In statistical terminology, the specific ARMA model we adopt is known as an ARMA(*p, d, q*) process, where *p, d* and *q* determine the window length of the time series used to make predictions. With respect to our purposes, the *d* parameter decides whether the ARMA models relations between absolute INI durations (*d* = 0) or instead between *relative* durations of adjacent INIs (*d* = 1). This is known as the degree of *differencing* in the series: to answer our question (i) above, we ask whether the model captures the series better with *d* = 0 or with *d* = 1. Models with *d* > 1 are possible, but the psychological interpretation of higher-order differencing is not straightforward, so we do not consider those models here. The parameters *p* and *q* determine how far back past the current to-be-predicted interval the model looks when calculating its prediction, which corresponds to the *order* in what we have been calling “higher-order” structure. The model computes predictions in two ways: by computing an “autoregressive” component and a “moving average” component. The standard technical details of this model are rigorously explained in the literature (Jones, 1980; Hamilton, 1994); it is sufficient to note that *p* and *q* determine how far back the model looks in these calculations respectively: the model performs autoregression on the *p* previous intervals, and calculates a moving average component for *q* previous intervals. Quantifying higher-order predictability corresponds to asking what combination of *p* and *q* (and *d*) lead to the most accurate model predictions. If the model makes better predictions by seeing more steps backward (controlling for increased model complexity, see below), this indicates the existence of predictability at higher-order. In principle *p* and *q* can grow unboundedly, but for reasons of practicality we impose a maximum depth on these parameters: specifically, the space of models we search is subject to the constraint *p, q* ≤ 5 (and *d* ≤ 1). In other words, we consider all ARMA models up to an order of five, where the order is the total number of previous durational observations taken into account.

### Akaike weights: ranking models based on their parsimony and fit to the data

We use the R library “forecast” (Hyndman and Khandakar, 2008; R Core Team, 2013) to fit the ARMA models to our data. This library can handle the missing INIs across phrase breaks, and does so by maximizing the likelihood of the model given all data that is present. Additionally, the preceding difference in intensity was fit by the ARMA model as an *external regressor*, adding this first-order intensity difference to the linear model. Then, for each language, we identified the model with the lowest AICc value (Akaike Information Criterion, Burnham and Anderson, 2002) as the one that fit our data the best. The AIC is the most common criterion to perform model selection in ARMA models (Brockwell and Davis, 1991): intuitively, AIC provides a score that reflects how well a model captures the data, whilst also penalizing model complexity. AICc corrects this measure for small sample sizes.

While AICc scores correct for model complexity, more complex operations such as addition, taking a mean or comparison of groups of models cannot be performed meaningfully using these values alone. Wagenmakers and Farrell (2004) describe how to calculate *Akaike weights*, which allow for a more advanced quantitative comparison between models. More specifically, the Akaike weights *w*_*i*_ are a measure of a model's predictive power relative to the combined predictive power of all models considered, and can be calculated over a collection of AICc scores *AICc*_*j*_ as follows:

$$\begin{array}{lll} {ŵ}_{i} & = & \text{exp}\left(-\frac{1}{2}\left(AICc_{i}-{\text{min}}_{j}\left(AICc_{j}\right)\right)\right)\\ w_{i} & = & \frac{{ŵ}_{i}}{\underset{j}{\sum }{ŵ}_{j}} \end{array}$$

Using these weights (which sum up to a total of 1), we identified the *Akaike set*: the set of all highest-ranked models summing up to a cumulative Akaike weight of at least 0.95 (Johnson and Omland, 2004; Ravignani et al., 2015), in order to provide a view on the robustness of the best-fitting model. By aggregating the Akaike weights in this way, we (i) gain the combined explanatory power of multiple models instead of just the best one, and (ii) counteract the volatility of the analysis: i.e., if there are relatively few models with a high Akaike weight in this Akaike set, and most of them share a particular feature, we have more confidence in the importance of this feature than by just exploring the single best model.

In our particular analysis, we can test the hypotheses above by observing how Akaike weight is spread across the 72 different model variants: the larger the weight taken up by the relevant subset of models (i.e., with *p* above zero, or with *d* = 1) in the Akaike set, the stronger the support for the hypothesis. In sum, these techniques allow us to judge the features of models that explain the data well, while favoring simpler models, and without the need to choose a single best candidate.

**Figure 4:**
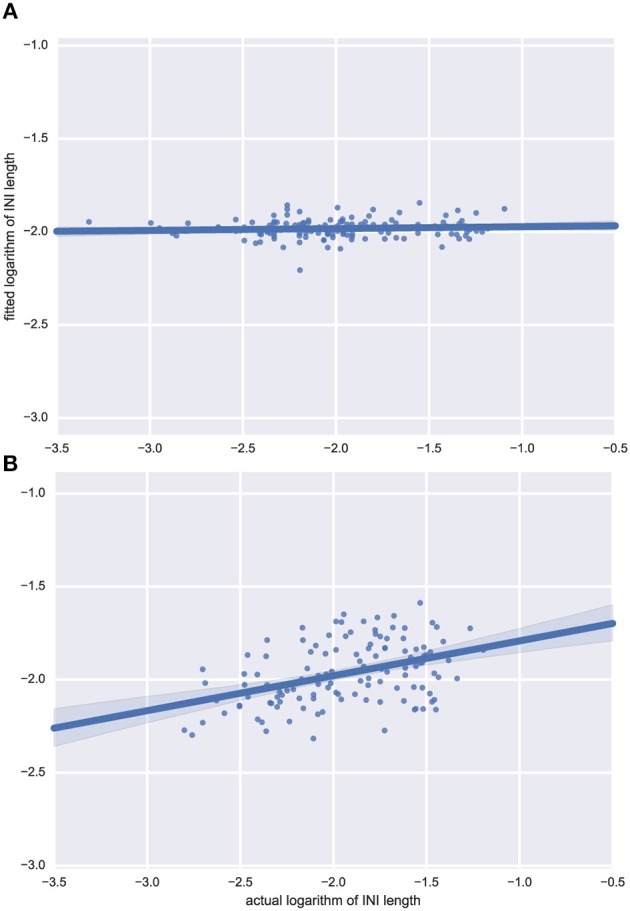
**Examples of model predictions for two languages**. Fitted model predictions for **(A)** Japanese and **(B)** Dutch, showing that even one of the best fits amongst all languages, Dutch, only captures a limited amount of temporal structure. For some other languages (such as Japanese) ARMA does not manage to make better predictions than values slightly varying around the mean INI. Actual data points (logarithms of INI durations) on the x-axis, vs. predictions of the fitted model on the y-axis; perfect predictions would correspond to a diagonal (45°) line.

Inference in time-series analysis is notoriously volatile, especially for small sample sizes and for series that include missing values. In our case, these missing values are derived from phrase and sentence breaks and other disruptions to the speech rhythm. This was clear in our results: although the range of ARMA-based analyses we pursued did consistently outperform baseline random-noise based alternatives, it did not lead to strong inferences: even the best-fitting ARMA models explained only a small portion of the data. Figures 4A,B respectively show examples of bad and good fit to our data. We therefore report results over a variety of possible models, an approach known as *multi-model inference* which smoothes over uncertainty in model selection.

### Results of the time series analysis on nuclei timing

First, to address question (i) above, we compared the combined Akaike weight, for each language, of models which represented the data as *relative* durations vs. absolute durations. Relative durations models (i.e., *d* = 1) have a notably high sum of Akaike weights, for almost all languages (see Table 1 and Figure 3B). This suggests that the model is most powerful when looking at the data as (the logarithms of the) *relative* durations. This is intuitive from a psychological perspective, both in terms of the log-scaling, and in terms of the focus on relative durations rather than absolute temporal duration (Grondin, 2010; McAuley, 2010).

Second, we accumulated the Akaike weights of all models with the same value for *p*; we then compared these marginal Akaike sums over *q* and *d* between *p*, as a way of investigating the importance of the autoregressive component's order. The higher *p* is, the more time-steps backwards the AR component of the ARMA model can use in order to predict where the next syllable nucleus will occur. As such, the extent to which the combined Akaike weights for larger values of *p* exceed the equivalent weights for *p* = 0 or *p* = 1 provides a window onto higher-order structure: more specifically, an indication of how well higher-order regularities and patterns in our data are captured by the ARMA model. A higher order in the moving average portion of the model, determined by *q*, is less important because the MA process only captures temporal dependencies in the random error. That is, the MA can explain some variance attributable to e.g., drift in INI length (for instance, when speaking rate increases or decreases over time), but does not have a straightforward correlate in terms of predictability of syllable nuclei. As such we focus on *p* as an indicator of higher-order predictability, marginalizing over *q*.

Figure 3C depicts the *marginalized Akaike weights* (i.e., weights summed over possible values for *d* and *q*) for each *p* and language. As can be seen, this visualization reveals a less clear picture of the distribution of the Akaike weights. AICc quantifies the quality of a fit while taking in account a penalty for model complexity. Hence, a partition of identical weights for each *p* and language would be the least informative with respect to the best order of the model. Instead, if the higher-order dependencies were adding nothing at all to the model's predictive power, we would expect the Akaike weights to be concentrated strongly on just *p* = 0. Likewise, if higher-order dependencies made improvements to the model's predictions, we would expect one or some of the *p* > 0 models to reserve positive Akaike weight.

Figure 3C reveals a subtle pattern of results. On the one hand, we see that for most languages, Akaike weight is concentrated on lower-order models (*p* = 0, *p* = 1), arguing against the idea that higher-order dependencies make dramatic improvements to prediction (under the assumptions of the ARMA model). On the other hand, even among these cases, higher-order models often still reserve *some* Akaike weight, even after being penalized for increased complexity. This suggests that higher-order models may still be capturing meaningful structure, even where lower-order dependencies are more powerful predictors. Moreover, there are some notable cases, such as Dutch, Mapudungun, and Turkish, in which higher-order models reserve extremely strong Akaike weight, at the expense of lower order models. This suggests that in these cases, models which are able to capture temporal dependencies at higher orders represent our best description of the data.

### Discussion: what can time series tell us about speech timing?

Overall, the ARMA analysis hints at the possibility that temporal regularities exist at higher orders in at least some of our data. We take this as strong motivation to explore the possibility further in future work, but hesitate to draw strong conclusions given the limitations on the models' predictive power and the variability in results across languages. In this respect our findings mirror previous results on rhythmical structures in speech, which have also often not led to strong conclusions, and demonstrated sensitivity to idiosyncrasies of the data (Arvaniti, 2012). A conservative conclusion is that, even if there is predictability at higher orders, only some of this structure appears capturable by the ARMA analysis we undertook. This could have multiple reasons, ranging from idiosyncrasies of our data and our statistical approach, to more general questions about the presence and nexus of temporal structure in speech, as follows:

- The amount of data per language may not be enough to give clear results,
- The linear ARMA models may be theoretically unable to fit and predict the timings of our syllable nuclei,
- The structural form of the ARMA model is better suited to capture the regularities in speech at lower-order, whereas higher-order regularities that exist in these languages take a form that the ARMA model cannot fully capitalize on in its predictions, for instance fractal long-range correlations (Schenkel et al., 1993; Levitin et al., 2012; Delignières and Marmelat, 2013),
- The features we extracted from the speech data (i.e., INI lengths and intensity) may be too limited and provide no clear patterns, or
- There may well be no complex structures to be found in speech that provide considerably more predictability than simple zero- or first-order measures.

Deciding between these possibilities is a clear objective for future research. A natural starting point would be to work with more data (i) or different data (i.e., different features, iv): either more data per language, or more data from a subset of languages, or data from multiple speakers per language. Another approach would be to look in more detail at ARMA predictions, and perhaps consider generalizations or more complex time-series models that build on or relax some of the assumptions in the classic ARMA (e.g., the linearity assumption, ii, iii) Such models exist and could be explored in our data, or new data. The final possibility (v), that there are no structures to be found at this level, could only be upheld by ruling out possibilities i–iv, which our analyses cannot do.

Together, our analyses provide reasonable evidence for first or minimal order temporal structure (i.e., for the role of relative durations in the perception of rhythm in speech), and weaker evidence for principled higher-order structure that can be captured by linear regression models such as ARMA.

## General discussion and conclusions

Temporal structure is a central aspect of speech processing. Multiple studies have shown that infants rely on the rhythm type of their native language as a guide for speech segmentation (Nazzi and Ramus, 2003; Saffran et al., 2006). The extent to which higher-order sequences are used in predicting subsequent events or INIs is debated. Humans perform poorly at detecting temporal structure in mildly complex patterns (Cope et al., 2012). Finding regularity across a number of intervals correlates with reading ability, while detecting gradual speeding-up/slowing-down does not (Grube et al., 2014). However, to the best of our knowledge, no studies have ever provided a quantitative analysis of how the temporal properties of the speech signal determines predictability within the speech signal. Does the temporal structure of our data portray regularities that allow the duration and location of upcoming syllables to be predicted? Our approach to this question was 2-fold.

### Our approach: alternative metrics for low order temporal regularities

First, in line with many other studies (Arvaniti, 2012), we focused on lower order temporal regularity. Existing metrics for speech rhythm at this level of analysis tend to be applied to research objectives that are slightly different to ours (e.g., classifying languages into rhythmic groups), and have been shown to be somewhat unreliable in the sense that they are often sensitive to idiosyncrasies of the data they model. In this light, our lower-order analyses focused first on maximal simplicity, then on quantifying predictability from the perspective of an ideal observer. These approaches proved useful for quantification of predictability at this level, showing broad support for constrained, but not complete regularity in INIs across the languages in our sample. These results are in keeping with the general and well-attested idea that there is temporal regularity in syllable timing, but that this regularity is not sufficient to account for the subjective experience of rhythm in speech (Lehiste, 1977). We add to this insight that a similar ceiling appears to also constrain how well these lower-order regularities can aid speech segmentation and acquisition in terms of predictability.

### Our approach: introducing time series analysis to speech timing

Second, we tried to quantify predictability that might exist at higher-order temporal resolution in our dataset, a topic that, to the best of our knowledge, has received little attention in previous work^1^. We chose to model INI sequences as time-series, and to make inferences about the order of dependencies in those series through model-fitting. This approach is a natural generalization of existing lower-order metrics: it allowed us to leverage a range of tried-and-tested methods of analysis in spite of the complexity inherent to higher-order forecasting. However, the results of our analyses provide only weak support for higher-order predictability. We highlighted a range of possible reasons for this above. Naturally, it is possible that our data are unsuited to the problem, or that our inferential methods were simply not powerful enough given the data. We disfavor this possibility for all the reasons discussed in the introduction and materials and methods. An alternative conclusion is that these regularities are not there to be found at higher orders. Again, we are hesitant of this conclusion, though acknowledge that it may chime with what others have claimed about speech rhythm in general (see Lehiste, 1977). The ARMA model, while widely used and a natural first contender, may be inherently unable to capture this important, though yet unknown, class of regularities: in particular, the ARMA model can only make predictions about the future on the basis of *linear* combinations of the past, which may be too restrictive.

### Alternative hypotheses: is predictability contained in the speech signal, or is predictability a top-down cognitive trait?

An alternative explanation is that few regularities exist but humans hear rhythmic patterns in speech because they impose top-down expectations: for instance, humans perceive time intervals as more regular than they really are (Scott et al., 1985) and impose metric alterations to sequences which are physically identical (Brochard et al., 2003). However, exposure to strong temporal irregularities can make humans perceive regular events as irregular (Rhodes and Di Luca, 2016). Mildly regular—predictable though non-isochronous—patterns are perceived quite well, possibly based on local properties of the pattern (Cope et al., 2012). In any case it seems that human perception of rhythm is not simply a matter of determining time intervals between acoustic intensity peaks, but that it involves a more complex process, potentially integrating multiple prosodic cues such as pitch, duration, INI or intensity values.

Top-down and global/local regularity perception relates to the question of whether the ability to perceive and entrain to temporal patterns in speech may benefit language processing at both a developmental and an evolutionary scale. From an evolutionary perspective, overregularization of perceived patterns combined with mild regularities in the speech signal might hint at culture-biology co-evolutionary processes. It would suggest that humans might have developed top-down mechanisms to regularize highly variable speech signals, which would have in turn acquired slightly more regularities (for biology-culture coevolution in language and speech, see: Perlman et al., 2014; de Boer, 2016; Thompson et al., 2016).

### Future work

All the analyses above are based on only one speaker per language. Having multiple speakers for each language would have been preferable to account for speaker variability; Ideally, 18 speakers per language (as many as the languages encompassed in this study), would have allowed a meta-analysis via a 18 × 18 repeated measures ANOVA to test whether most variance could be explained by the language or rather the speaker/annotator factor. However, as we neither find, nor claim, existence of categorical differences between languages, we believe speaker variability is not an issue in the current analysis. Had we found strong differences between languages, we would not be able to know—with only one speaker per language—whether these were due to a particular language or, rather, to the particular speaker of that language. On the contrary, all our results are quite similar across languages and, importantly, annotators. The few outliers (Cantonese, Hungarian, and Turkish) should be investigated in future research by having many speakers and many annotators for each of them. Ours is in fact just a first attempt at introducing the Bayesian and time series approaches to the world of speech timing.

While annotating the language samples, we did not use pre-conceived notions about the building blocks of speech based on writing systems. Rather, we used clearly defined acoustic measures to define the events. Our approach is supported by evidence from analysis of phonological processes showing that syllables have cognitive reality even without writing. Moreover, although the sample size was small, our statistical methods were shown in the past powerful enough for comparable sample sizes, and for our sample could detect *some* regularities. Future studies with larger samples will test if analyzing more languages, or longer samples per language, leaves our controversial results unvaried. Should a replication confirm our negative result, this would suggest that the effect size of temporal predictability of speech is so small that it is unlikely to play an important role in the acquisition of speech.

We suggest that the ARMA model we use here to model syllable timing could be used to model another aspect of speech rhythm, namely amplitude modulation. It has been suggested that modulation in the envelope of the speech signal at different time scales might provide a useful physical correlate to rhythm perception (Goswami and Leong, 2013). In particular, the timing of signal amplitude decrease/increase and phase difference between modulation rates at different scales within the same speech signal might encode much rhythmic information (Goswami and Leong, 2013), which is not captured by our temporal prediction model above. However, hypotheses on predictability in amplitude modulation could be tested across languages using the same time series approach we use here. By swapping the roles of intensity and duration in the model above, one would allow a range of past intensity values to predict the timing and intensity of the upcoming syllable. High lag order of the resulting amplitude-modulation ARMA, possibly together with a lower Akaike than our time prediction model, would provide empirical support for the amplitude modulation hypothesis.

Further comparative research on temporal structure perception in speech with nonhuman animal species could better inform our understanding of the evolutionary path of such an ability, determining how much this ability depends on general pattern learning processes vs. speech-specific combination of cues (Ramus et al., 2000; Toro et al., 2003; Patel, 2006; Fitch, 2012; de la Mora et al., 2013; Ravignani et al., 2014; Spierings and ten Cate, 2014; Hoeschele and Fitch, 2016).

Finally, alternative algorithms and toolboxes could be tested and compared to our manual annotation results. Crucial desiderata for such algorithms are to: (1) yield more robust results than the unsatisfying automated approaches which spurred our manual annotation in the first place; (2) be at least as psychologically plausible as our manual annotation; (3) work properly across different language families and phonological patterns. These desiderata might be partially or fully satisfied by using and adapting algorithms originally developed for music analysis. In particular, interesting research directions at the boundary between experimental psychology and artificial intelligence could be: (i) performing automated annotations after adapting the “tempogram toolbox” (Grosche and Muller, 2011) to the speech signal, (ii) assessing the perceptual plausibility of the beat histogram (Lykartsis and Weinzierl, 2015) and the empirical mode decomposition of the speech amplitude envelope (Tilsen and Arvaniti, 2013), and (iii) further testing beat tracking algorithms already used in speech turn-taking (Schultz et al., 2016).

### Conclusions

Taken together, what do our analyses imply about the existence and locus of temporal predictability in speech? Others have argued that subjectively-perceived rhythm in speech may result from coupled or hierarchical series of events at multiple timescales *across* domains in speech (e.g., Cummins and Port, 1998; Tilsen, 2009). Our results speak only to predictability in the temporal relations between syllables. Nevertheless, these results hint at a broadly complementary perspective: *within* one domain, regularity in temporal structure is difficult (but not impossible) to capture with our methods, suggesting that the degree of predictability available to a learner is weak or unreliable at any individual level (e.g., first order, second order regularities). However, the following hypothesis strikes us as worthy of investigation in a statistical framework: the impression of regularity and predictability may result from the *combination* of cues at multiple levels, even though individually these cues may be weak.

Our results somewhat undermine a simplistic view of the usefulness of rhythm in language acquisition (Pompino-Marschall, 1988). Future research should further investigate the interaction of acoustic features underlying the perception of phonological patterns in natural languages. Research along these lines will improve our understanding of the interplay between predictability and learning, informing the debate on both language acquisition and language evolution.

## Overview of the data files and their formats

### Raw annotations

The data is available as Supplementary Material and at: https://10.6084/m9.figshare.3495710.v1.

The files with extension .zip, having the format Language_iso_annotator.zip contain the raw annotations in a saved Praat TextGrid. They annotate the narrative sound files of the Illustrations of the IPA, as provided by the *Journal of the International Phonetics Association* (https://www.internationalphoneticassociation.org/content/journal-ipa). Whenever this audio data consisted of multiple files, multiple Praat files with annotation were created.

These annotations also contain the perceived phrase and sentence breaks (respectively by a / and // marker), that interrupted the sequences of contiguously uttered speech.

The individual TextGrid files should all be readable by Praat, version 6.

### Prepared data

The previously mentioned TextGrid annotations were enhanced by adding the intensities and were then converted into a format that was easier to read by our analyses scripts. The Language_iso_annotator.out files are tab-separated text files that contains 4 columns, with each row corresponding to a single syllable nucleus annotation:

- The first column, part, refers to the order of the audio files of the narrative.
- The second column, time, refers to the location in the audio file the annotation was added.
- The third column, mark, can be empty or can contain the / or // symbols, indicating a phrase or sentence break.
- The fourth column, intensity, shows the intensity of the audio recording at the specified point in time, as calculated by Praat.

all.out assembles the previously described data from all different languages, while all_unique.out contains the data of only one annotator for each language. To distinguish between the different concatenated datasets, these two tab-separated files contain 2 extra columns:

- language contains the ISO code per language (cfr. the second part of the previous filenames).
- annotator contains the initials identifying the author that created this annotation (cfr. the third part of the previous filenames).

### Python conversion scripts

The Python script files (.py extension) are the ones that were used to convert the Praat .TextGrid format to the tab-separated .out files. They are included as a reference for the interested, but will not be executable as they depend on a self-created (and for now unfinished and unreleased) Python library to extract the intensities with Praat. Feel free to contact the authors for further explanation or access to the analysis scripts.

## Author contributions

BdB conceived the research, YJ, BT, PF, and BdB annotated the language recordings, YJ, AR, and BT analyzed the data. All authors wrote the manuscript.

## Funding

This research was supported by European Research Council grant 283435 ABACUS to BdB, and by a PhD Fellowship (Aspirant) of the Research Foundation Flanders - Vlaanderen (FWO) to YJ.

### Conflict of interest statement

The authors declare that the research was conducted in the absence of any commercial or financial relationships that could be construed as a potential conflict of interest.

## Footnotes

^1^Though see Liss et al. (2010), who also examine higher-order dependencies; in particular, they used the spectrum of the intensity envelope to recognize dysarthrias in speech, a condition resulting in the perception of “disturbed speech rhythm”: since peaks in the spectrum represent a linear relationship within the original time domain, ARMA could potentially capture the same kind of structure, though the ultimate goal of our article is different.
